# Investigating the association of pan-immune-inflammation value, systemic immune-inflammation index, and neutrophil-to-lymphocyte ratio with pain in Parkinson’s disease

**DOI:** 10.3389/fneur.2025.1682964

**Published:** 2025-10-29

**Authors:** Heyue Pan, Xiaohua Wang, Xiulin Zhang, Xiangsong Shi, Taipeng Sun, Jianyang Xu, Shouyong Wang

**Affiliations:** ^1^Huai’An No. 2 Clinical College of Xuzhou Medical University, Huai’an, China; ^2^Department of Neurology, Huai’An No. 3 People’s Hospital, Huai’an, China; ^3^Department of Medical Psychology, Huai’An No. 3 People’s Hospital, Huai’an, China

**Keywords:** Parkinson’s disease, pain, KING’s Parkinson’s Disease Pain Scale (KPPS), novel composite inflammatory markers, pan-immune-inflammation value, systemic immune-inflammation index

## Abstract

**Objective:**

To explore the relationship between novel composite inflammatory markers—pan-immune-inflammation value (PIV), systemic immune-inflammation index (SII), and neutrophil-to-lymphocyte ratio (NLR)—and the presence of pain in patients with Parkinson’s disease (PD).

**Methods:**

A total of 150 PD patients who attended the outpatient or inpatient departments of the Second Clinical College of Xuzhou Medical University (Huai’an Third People’s Hospital) between September 2020 and December 2023 were enrolled as the PD group. An additional 150 age- and sex-matched healthy individuals undergoing routine physical examinations were selected as the healthy control (HC) group. The King’s Parkinson’s Disease Pain Scale (KPPS), Hoehn-Yahr (H-Y) staging, Unified Parkinson’s Disease Rating Scale part III (UPDRS-III), Hamilton Depression Rating Scale-24 (HAMD-24), and Hamilton Anxiety Rating Scale (HAMA) were used to assess pain, disease severity, motor symptoms, depression, and anxiety in PD patients. Demographic and laboratory data were collected for all participants. Based on KPPS scores, PD patients were subdivided into those with pain (PDP group) and those without pain (nPDP group). Intergroup differences were compared, and the associations of PIV, SII, and NLR with pain in PD were analyzed.

**Results:**

Among the 150 PD patients, 79 (52.7%) reported pain, with a mean KPPS score of 10.81 ± 8.67. Compared to the HC group, PD patients exhibited significantly elevated levels of PIV, SII/100, and NLR, and significantly lower platelet and lymphocyte counts (*p* < 0.05). In subgroup analysis, PIV, SII/100, H-Y stage, UPDRS-III, and HAMD-24 scores were significantly higher in the PDP group than in the nPDP group (*p* < 0.05). KPPS scores were positively correlated with PIV, H-Y stage, UPDRS-III, and HAMD-24 scores, but not with SII/100 or NLR.

**Conclusion:**

Inflammatory dysregulation is present in PD patients. Compared with the nPDP group, patients in the PDP group showed significantly higher levels of PIV and SII/100, as well as greater disease severity (H-Y stage, UPDRS-III) and more pronounced depressive symptoms (HAMD-24) (*p* < 0.05). Moreover, KPPS scores in PD patients were not only associated with PIV but also positively correlated with disease stage, motor function impairment, overall disease severity, anxiety, and depression.

## 1 Introduction

Parkinson’s disease (PD) is a common neurodegenerative disorder primarily characterized by motor symptoms such as tremor, rigidity and bradykinesia. In addition to motor impairments, non-motor symptoms—including olfactory dysfunction, pain, and mood disturbances—are also frequently observed and are of considerable clinical importance. Among these, pain affects approximately 40 to 85% of PD patients (1) and has been shown to exert a substantial negative impact on patients’ mental health and quality of life (2), potentially leading to disability in severe cases. The mechanisms underlying pain in PD remain unclear. Recent studies (3, 4) suggest that neuroinflammation, dysregulation of neurotransmitters involved in nociceptive transmission, and a reduced pain threshold may all contribute to the development of pain in PD. Inflammatory cytokines have been extensively studied in PD animal models, and elevated levels of pro-inflammatory cytokines in the peripheral blood of PD patients (5) further support the notion that peripheral inflammation is closely associated with PD pathophysiology. Research has demonstrated a strong link between inflammation and non-motor symptoms in PD, with cognitive impairment being particularly relevant (6). In recent years, several novel composite inflammatory markers have emerged as promising tools for assessing systemic immune status, including the pan-immune-inflammation value (PIV) (7), neutrophil-to-lymphocyte ratio (NLR), and systemic immune-inflammation index (SII) (8). For example, SII is calculated as neutrophil count × platelet count / lymphocyte count, reflecting the balance between systemic inflammation and immune response more comprehensively. These indices have been linked to disease severity and prognosis in various inflammatory-related conditions, such as IgA nephropathy, hidradenitis suppurativa, and melanoma (9–11). Previous studies have explored the association of these markers with non-motor symptoms in PD. Hao et al. (12) reported a significant association between SII and depression in PD patients. Similarly, research by Zhai et al. (13) found that SII and NLR were significantly associated with fatigue in PD. These findings suggest that composite inflammatory markers such as PIV may be relevant to non-motor symptoms in PD, particularly mood and fatigue. However, few studies have examined their relationship with PD-associated pain. Therefore, this study aims to investigate the potential association of PIV, SII, and NLR with pain in PD patients. By evaluating these novel inflammatory markers, we seek to identify potential biomarkers for PD-related pain and to provide new insights that may contribute to improved diagnosis and management of PD.

## 2 Materials and methods

### 2.1 General information

A total of 150 patients diagnosed with Parkinson’s disease (PD) and treated at the Second Clinical College of Xuzhou Medical University (Huai’an Third People’s Hospital) between September 2020 and December 2023 were enrolled as the PD group. Inclusion criteria were as follows: (1) Diagnosis of PD according to the 2016 Chinese diagnostic criteria for Parkinson’s disease (14); (2) Complete baseline demographic data and peripheral blood test results available. Exclusion criteria included: (1) Parkinson-plus syndromes or secondary parkinsonism caused by traumatic brain injury, cerebrovascular disease, etc.; (2) Cardiac, pulmonary, hepatic, or renal insufficiency, or hematological disorders; (3) Autoimmune diseases, use of immunosuppressive agents or corticosteroids, history of malignancy, or recent acute/chronic infections; (4) Cognitive impairment or psychiatric symptoms that impeded completion of clinical assessments.

Additionally, 150 age- and sex-matched healthy individuals undergoing routine physical examination during the same period were recruited as the healthy control (HC) group. Exclusion criteria for the HC group included: (1) Incomplete baseline data or blood test results; (2) Cardiac, pulmonary, hepatic, or renal insufficiency, or hematological disorders; (3) Autoimmune diseases, use of immunosuppressants or antibiotics, recent infections (acute or chronic), or malignancy.

This study was approved by the Ethics Committee of the Second Clinical College of Xuzhou Medical University (Huai’an Third People’s Hospital) (approval no. 2023-015). Informed consent was obtained from all patients or their legally authorized representatives.

### 2.2 Methods

#### 2.2.1 Collection of demographic and clinical data

Baseline demographic information, including age and sex, was collected for all participants. For individuals in the PD group, additional clinical data were recorded, including Hoehn-Yahr (H-Y) staging, Unified Parkinson’s Disease Rating Scale part III (UPDRS-III) scores, disease duration, King’s Parkinson’s Disease Pain Scale (KPPS) scores, and scores from the Hamilton Anxiety Rating Scale (HAMA) and Hamilton Depression Rating Scale-24 (HAMD-24).

#### 2.2.2 Laboratory assessments

Fasting peripheral venous blood samples were collected from all participants in the early morning. Complete blood counts were measured using an automated hematology analyzer (Mindray BC-7500CS). Immuno-inflammatory parameters were calculated based on the following measured components: neutrophil count (N), monocyte count (M), lymphocyte count (LYM), and platelet count (PLT). From these, the pan-immune-inflammation value (PIV), systemic immune-inflammation index (SII), and neutrophil-to-lymphocyte ratio (NLR) were derived according to standard formulas. Due to the large numerical range of SII, values were normalized as SII/100 to facilitate statistical interpretation without affecting significance.

#### 2.2.3 Clinical evaluation of motor and psychological status

Motor function and disease severity in PD patients were assessed using the Unified Parkinson’s Disease Rating Scale part III (UPDRS-III) and Hoehn-Yahr staging. All clinical assessments were performed independently by two board-certified neurologists while patients were in the “on” medication state. Depression was evaluated using the 24-item Hamilton Depression Rating Scale (HAMD-24), with scores ≥17 indicating clinically relevant depression. Anxiety was assessed using the Hamilton Anxiety Rating Scale (HAMA), with scores ≥14 indicating clinically significant anxiety.

#### 2.2.4 Pain assessment using the King’s Parkinson’s Disease Pain Scale (KPPS)

Pain was evaluated using the King’s Parkinson’s Disease Pain Scale (KPPS), which comprises 14 items, each scored on a scale from 0 to 12, yielding a total possible score ranging from 0 to 168. A score of ≥1 was considered indicative of the presence of pain, with higher scores reflecting greater pain severity. Based on KPPS scores, patients in the PD group were further categorized into those with pain (PDP group, *n* = 79) and those without pain (nPDP group, *n* = 71).

### 2.3 Statistical analysis

All statistical analyses and data visualizations were performed using SPSS version 26.0 (IBM Corp., Armonk, NY, United States) and R software version 4.3. Continuous variables were expressed as mean ± standard deviation (SD). Between-group comparisons of continuous variables were conducted using independent-sample t-tests, while categorical variables were compared using the chi-square (*χ^2^*) test. Correlations between KPPS scores (as a measure of pain severity in PD patients) and clinical parameters were assessed using Spearman’s rank correlation analysis. A two-tailed *p*-value < 0.05 was considered statistically significant.

## 3 Results

### 3.1 Comparison of baseline characteristics and clinical indicators between the PD and HC groups

A total of 300 participants were enrolled in this study, with 150 individuals in the PD group and 150 in the HC group. No significant differences were observed between the two groups in terms of sex distribution, age, monocyte count, or neutrophil count (*p* > 0.05). However, the levels of all three composite inflammatory markers were significantly elevated in the PD group relative to the HC group. Significant differences were found in PIV, SII/100, NLR, platelet count, and lymphocyte count (*p* < 0.05; Table 1).

**Table 1:** Comparison of baseline characteristics and clinical indicators between the PD and HC groups.

Characteristic	HC, *n* = 150	PD, *n* = 150	χ^2^/w/t	*p*-value
NLR, median (Q1, Q3)	1.65 (1.28, 2.09)	1.93 (1.48, 2.67)	8,232.00	<0.001
PIV, median (Q1, Q3)	112 (81, 160)	131 (90, 180)	9,266.00	0.008
SII/100, median (Q1, Q3)	3.69 (2.83, 4.73)	4.06 (2.93, 5.52)	9,598.50	0.028
Neutrophil count, median (Q1, Q3)	3.17 (2.72, 4.05)	3.01 (2.54, 3.84)	12,458.50	0.108
Age, median (Q1, Q3)	69 (65, 72)	68 (61, 73)	11,643.50	0.600
Lymphocyte count, median (Q1, Q3)	2.01 (1.66, 2.36)	1.60 (1.17, 1.98)	16,337.50	<0.001
Platelet count, mean ± SD	241 ± 50	198 ± 50	7.25	<0.001
Monocyte count, median (Q1, Q3)	0.32 (0.27, 0.40)	0.32 (0.25, 0.39)	11,447.00	0.794
Sex, *n* (%)			1.08	0.298
Female	75 (50.0%)	66 (44.0%)		
Male	75 (50.0%)	84 (56.0%)		

SII/100 = systemic immune-inflammation index divided by 100; NLR = neutrophil-to-lymphocyte ratio; PIV = pan-immune-inflammation value; HC = healthy control group; PD = Parkinson’s disease group.

### 3.2 Comparison between PD patients with and without pain

**Figure 1:**
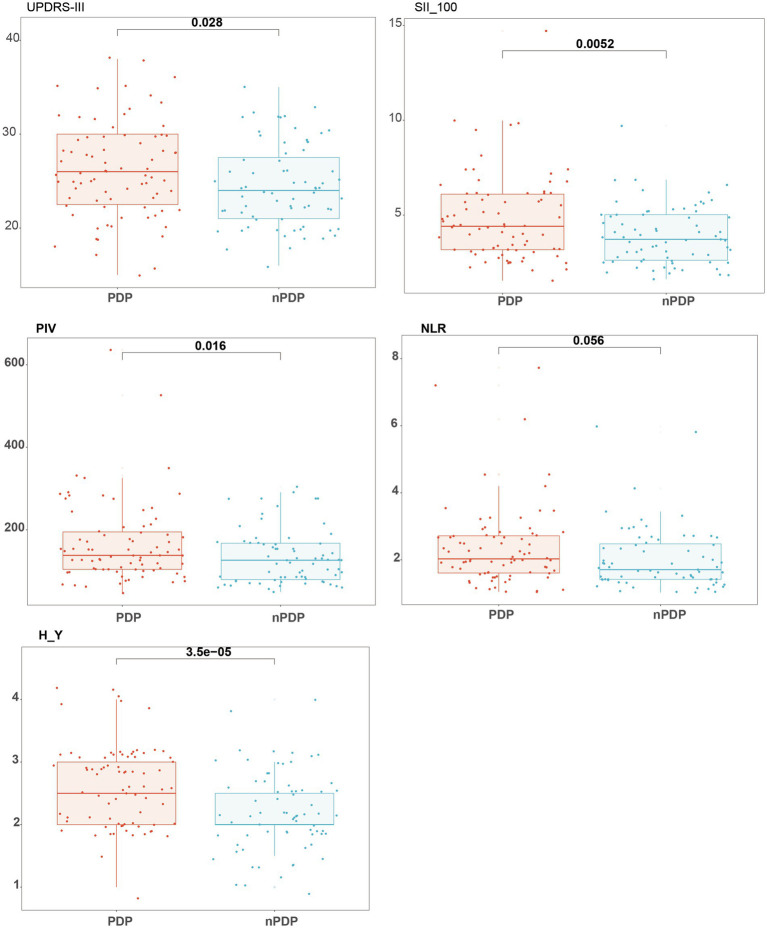
Between-group comparison of key clinical and inflammatory parameters in the PDP and nPDP groups. With = PDP group (PD patients with pain); without = nPDP group (PD patients without pain); H-Y = Hoehn-Yahr stage; UPDRS = Unified Parkinson’s Disease Rating Scale, part III.

Among the 150 patients in the PD group, 79 (52.7%) experienced pain (PDP group), including 33 females and 46 males, with a mean KPPS score of 10.81 ± 8.67. The remaining 71 patients (47.3%) were classified as the non-pain group (nPDP), comprising 37 females and 34 males. Significant differences were observed between the PDP and nPDP groups in PIV, SII/100, HAMD-24 scores, Hoehn-Yahr stage, and UPDRS-III scores (*p* < 0.05; Figure 1). However, no significant differences were found in age, sex, disease duration, or NLR (*p* > 0.05; Table 2).

**Table 2:** Comparison of demographic and clinical characteristics between PDP and nPDP groups.

Variable	PDP, *n* = 79	nPDP, *n* = 71	χ^2^/w	*p*
Age, median (Q1, Q3)	67 (60, 72)	69 (63, 74)	2,515.00	0.276
Course, median (Q1, Q3)	5.00 (3.00, 7.00)	5.00 (3.00, 7.00)	2,847.50	0.765
Sex, *n* (%)			1.61	0.205
Female	33 (41.8%)	37 (52.1%)		
Male	46 (58.2%)	34 (47.9%)		
Anxiety, *n* (%)			0.01	0.915
No	64 (81.0%)	58 (81.7%)		
Yes	15 (19.0%)	13 (18.3%)		
Depression, *n* (%)			5.66	0.017
No	55 (69.6%)	61 (85.9%)		
Yes	24 (30.4%)	10 (14.1%)		

PDP = Parkinson’s disease group with pain; nPDP = Parkinson’s disease group without pain.

### 3.3 Correlation between KPPS scores and clinical parameters in PD patients

In PD patients, KPPS scores were positively correlated with PIV, Hoehn-Yahr (H-Y) stage, UPDRS-III, HAMA, and HAMD-24 scores (*p* < 0.05), while no significant correlations were found with SII/100 or NLR. Detailed results are presented in Table 3.

**Table 3:** Correlation between KPPS scores and clinical parameters in PD patients.

Clinical indicators	r value	*p* value
NLR	0.21	0.059
PIV	0.32	0.005
SII/100	0.14	0.205
UPDRS-III	0.74	<0.001
H-Y stage	0.66	<0.001
HAMA	0.76	<0.001
HAMD-24	0.85	0.000

KPPS = King’s Parkinson’s Disease Pain Scale; HAMA = Hamilton Anxiety Rating Scale; HAMD-24 = 24-item Hamilton Depression Rating Scale.

## 4 Discussion

The etiology of PD remains uncertain, but increasing evidence supports a strong association between PD and inflammation. Prior studies have shown that levels of inflammatory cytokines such as IL-2, IL-6, IL-10, IFN-*γ*, IL-17A, and TNF are elevated in the peripheral blood of PD patients compared to healthy controls (15, 16). In addition, SII has been identified as an important factor associated with motor function in PD, with higher SII levels negatively correlated with scores on activities of daily living scales (17). The mechanisms underlying pain in PD are complex and not yet fully understood. Current evidence suggests that PD-related pain involves neuroinflammatory, sensory, and basal ganglia pathways (18). According to Ford’s classification, PD-related pain can be divided into musculoskeletal pain, central pain, neuropathic pain, dystonia-related pain, and akathisia (19). Despite its high prevalence, pain is often overlooked in clinical practice, and approximately 50% of PD patients with pain receive no specific analgesic treatment (20). KPPS is the first internationally validated scale specifically designed to assess pain in PD (21). In this study, KPPS was used to classify patients into PDP and nPDP groups, enabling the investigation of novel inflammatory markers such as PIV and SII in relation to pain.

Routine blood testing revealed significant differences in PIV, NLR, and SII/100 between the PD and HC groups (*p* < 0.05; Table 1), consistent with previous findings (15, 16). Additionally, lymphocyte and platelet counts differed significantly between groups. Although platelets are traditionally not considered central to inflammation, recent studies have highlighted their active role in immune regulation and inflammatory cascades, beyond their classic function in hemostasis (22). Between the PDP and nPDP groups, PIV and SII/100 were significantly higher in the PDP group, while NLR showed no significant difference. This may be due to the broader immunological profile captured by PIV and SII, which include monocytes and platelets in addition to neutrophils and lymphocytes, offering a more integrated reflection of systemic inflammatory status. Furthermore, the PDP group exhibited significantly higher H-Y stage, UPDRS-III, and HAMD-24 scores, suggesting that pain in PD is associated not only with inflammation, but also with more advanced disease stage, greater motor impairment, and more severe depressive symptoms (Figure 1). These results reinforce the hypothesis that PD-related pain is driven by a combination of pathophysiological and immune-inflammatory processes (23). While both PD pathogenesis and chronic pain are independently associated with inflammatory processes, the specific role of systemic inflammation in driving pain within PD remains unclear (24, 25). We hypothesize that the systemic immune dysregulation captured by PIV may contribute to PD-related pain through several pathways, including pro-inflammatory cytokine-mediated central sensitization, neuro-immune interactions facilitated by altered blood–brain barrier function, and the exacerbation of neurodegeneration under a chronic inflammatory state. Although our cross-sectional design cannot establish causality or a formal mediation effect, the distinct association of PIV with pain raises the possibility that systemic inflammation may serve as one pathophysiological link between PD and pain. This hypothesis warrants direct testing in future longitudinal studies designed to investigate the potential mediating role of inflammatory mechanisms. Although prior studies have indicated that sex may influence the prevalence and type of pain in PD (26, 27), no significant differences in age, sex, or disease duration were observed between the PDP and nPDP groups in our cohort, which may be attributable to the single-center design, sample size, or potential selection bias.

Among the composite inflammatory markers evaluated in this study, only PIV showed a significant correlation with pain severity in PD, while NLR and SII did not. This may be attributed to the fact that PIV is calculated using a combination of neutrophil count × monocyte count × platelet count/lymphocyte count, integrating four immune cell components. We speculate that this multi-parameter structure enables PIV to provide a more accurate and sensitive reflection of systemic immune-inflammatory responses, thereby enhancing its relevance to pain in PD. Beyond inflammatory markers, psychological factors such as depression and anxiety were also strongly associated with pain. In the subgroup comparison between PDP and nPDP, depressive symptoms differed significantly (Figure 1). Furthermore, correlation analysis showed that both HAMA and HAMD-24 scores were significantly associated with KPPS scores (Table 3). Previous studies have suggested a bidirectional relationship between pain and affective disorders, wherein anxiety and depression can enhance pain sensitivity, and psychological stress may exacerbate peripheral neuroinflammation, further intensifying localized pain (28). Accordingly, pain is often comorbid with affective disorders, and emotional disturbances are known to amplify pain perception (29). This study also found that PD-related pain was positively associated with H-Y stage and UPDRS-III scores (*p* < 0.001), indicating that more advanced disease stage and greater motor impairment are risk factors for pain. As H-Y stage increases, disease severity and motor dysfunction worsen, which may lead to heightened central sensitization and increased pain perception. Our findings confirm that H-Y stage, UPDRS-III score, and depressive symptoms not only differ between the PDP and nPDP groups but also correlate significantly with KPPS scores, in agreement with previous studies.

Taken together, we propose that PIV and, to a lesser extent, SII may serve as novel, noninvasive, and readily obtainable inflammatory biomarkers for identifying and evaluating PD patients with pain. This interpretation should be considered in the context of the study’s limitations. Its single-center, cross-sectional design may limit the generalizability of the findings and precludes causal inference. Furthermore, the assessment of inflammatory markers at a single time point (admission) does not reflect their dynamic changes throughout the disease course. Finally, the exploratory nature of this study meant that we did not perform advanced statistical corrections (e.g., FDR) or adjust for potential confounders such as sex and disease duration in the correlation analyses, which should be addressed in future validation studies with larger samples, prospective designs, and detailed pain phenotyping to further validate our findings.

## Data Availability

The original contributions presented in the study are included in the article/supplementary material, further inquiries can be directed to the corresponding author.
